# Vitamin D Status Presents Different Relationships with Severity in Metabolic-Associated Fatty Liver Disease Patients with or without Hepatitis B Infection

**DOI:** 10.3390/nu14102114

**Published:** 2022-05-18

**Authors:** Ling Luo, Junzhao Ye, Congxiang Shao, Yansong Lin, Yanhong Sun, Shiting Feng, Wei Wang, Bihui Zhong

**Affiliations:** 1 Department of Gastroenterology of the First Affiliated Hospital, Sun Yat-sen University, Guangzhou 510080, China; luol27@mail2.sysu.edu.cn (L.L.); yejzh@mail2.sysu.edu.cn (J.Y.); shaocx@mail2.sysu.edu.cn (C.S.); linys3@mail2.sysu.edu.cn (Y.L.); 2 Department of Clinical Laboratory, The East Division of the First Affiliated Hospital, Sun Yat-sen University, Guangzhou 510080, China; sunyh@mail.sysu.edu.cn; 3 Department of Radiology of the First Affiliated Hospital, Sun Yat-sen University, Guangzhou 510080, China; fengsht@mail.sysu.edu.cn; 4 Department of Medical Ultrasonics of the First Affiliated Hospital, Sun Yat-sen University, Guangzhou 510080, China; wangw73@mail.sysu.edu.cn

**Keywords:** vitamin D, liver fat content, metabolic-associated fatty liver disease, chronic hepatitis B, overlap

## Abstract

Whether the associations between serum vitamin D (VitD) and metabolic-associated fatty liver disease (MAFLD) vary with chronic hepatitis B (CHB) infection has not been well established. This study aims to investigate the relationships between serum VitD and metabolism, liver fat content (LFC) and fibrosis among MAFLD patients with and without CHB. Consecutive subjects (healthy controls: 360, CHB: 684, MAFLD: 521, CHB with MAFLD: 206) were prospectively enrolled between January 2015 and December 2021. Anthropometric, laboratory, imaging, and histological evaluations were conducted, with LFC measured via magnetic resonance imaging-based proton density fat fraction (MRI-PDFF). Serum VitD levels were lower in MAFLD patients than in healthy controls and patients with CHB alone or overlapping with MAFLD (24.4 ± 8.1 vs. 29.0 ± 9.5 vs. 27.4 ± 9.6 vs. 26.8 ± 8.4 ng/mL respectively; *p* < 0.001 in one-way ANOVA test). After adjusting for confounding factors, including season, hypersensitive C-reactive protein, insulin resistance, liver stiffness measurements, sun exposure, exercise and dietary intake, multivariate linear regression analysis revealed that VitD remained significantly negatively correlated with LFC in MAFLD patients (*β* = −0.38, *p* < 0.001), but not in CHB with MAFLD patients. Moreover, quantile regression models also demonstrated that lower VitD tertiles were inversely associated with the risk of insulin resistance and moderate–severe steatosis in the MAFLD group (*p* for trend <0.05) but not in the MAFLD with CHB group. VitD deficiency was associated with the severity of metabolic abnormalities and steatosis independent of lifestyle factors in MAFLD-alone subjects but not in MAFLD with CHB subjects.

## 1. Introduction

Metabolic-associated fatty liver disease (MAFLD), renamed from nonalcoholic fatty liver disease (NAFLD) by international expert consensus since July 2020 [1], and chronic hepatitis B (CHB) are two major chronic liver diseases worldwide [2]. With the growing obesity epidemic, the prevalence of MAFLD continues to increase, and has been reported to be as high as 33.0% in the general population [3]. Although universal vaccination programs have been implemented, CHB infection still affects over 240 million people globally [4]. Notably, the coexistence of MALFD and CHB is frequently observed, with an estimated MALD prevalence of 30% among subjects with CHB.

Vitamin D (VitD) is a pleiotropic steroid hormone that is well known for its role in the regulation of calcium homeostasis and bone mineralization. In serum, it exists as 25-hydroxyVitD (25(OH)D), which originates from the intake of certain foods and biosynthesis in the skin that relies on ultraviolet light exposure (the latter accounting for 80–90%). Several recent studies have implicated VitD deficiency in obesity, insulin resistance (IR), hepatitis B virus (HBV) replication and immune inflammation-related diseases [5,6,7]. A growing body of studies has paid attention to the relationship between VitD and chronic liver diseases, especially its relationship with MAFLD [8]. A recent meta-analysis, including 15 studies of 20,096 subjects (7803 NAFLD patients), confirmed that the levels of VitD in NAFLD subjects were approximately 15.4% lower than those in the controls (pooled VitD: 25.3 ± 13.7 vs. 29.1 ± 12.4 ng/mL, standardized mean differences: −0.90, 95% confident interval (CI): −1.29, −0.52) [9]. Additionally, another meta-analysis of 10 randomized controlled trials (544 NAFLD subjects) concerning the benefit of supplemental VitD in NAFLD found that VitD improved the levels of glycemic control and liver injury markers, including serum fasting glucose (FBG), homeostasis model assessment of insulin resistance (HOMA-IR), alanine aminotransferase (ALT) and triglyceride [10]. Moreover, a previous meta-analysis of seven studies with 814 CHB patients suggested that VitD levels were negatively correlated with HBV DNA levels: The pooled r (95% CI) was −0.41 (95% CI: −0.54, −0.27) [7]. A prospective cohort study found that CHB patients with VitD deficiency were associated with worse clinical outcomes [11]. However, there is little current evidence about the impact of VitD deficiency status on disease severity when MAFLD co-occurs with CHB.

In the present study, we aimed to compare the VitD levels among subjects with CHB, MAFLD and the co-incidence of the two, to explore the association among VitD, IR, liver fat content (LFC) and liver fibrosis in MAFLD patients with or without CHB, and to clarify whether their relationships were independent of lifestyle.

## 2. Materials and Methods

### 2.1. Study Population and Design

This was a prospective single-center, cross-sectional study conducted at the First Affiliated Hospital of Sun Yat-sen University (Guangzhou, China) from January 2015 to December 2021. The study plan was approved by the institutional ethics committee of The First Affiliated Hospital, Sun Yat-sen University (code: [2020]187) and written consent was obtained before enrollment.

We consecutively recruited patients from the Hepatology Outpatient Clinic, with the following inclusion criteria: (a) age ≥18 years; (b) diagnosis of CHB and/or MAFLD; (c) completion of anthropometric, laboratory and imaging data; and (d) no antiviral therapy or treatment for metabolic disorders. CHB was diagnosed in patients who displayed positivity for serum HBV DNA for over 6 months [12]. MAFLD was diagnosed according to the criteria approved by an international expert panel [13]. Patients who suffered from two kinds of diseases for more than 6 months were included in the MAFLD with CHB group. Other causes of liver disease, such as alcohol abuse (>210/140 g weekly for males/females), drug-induced liver injury, autoimmune hepatitis and other viral hepatitis were excluded. As a reference group, sex- and age-matched liver-healthy control subjects were selected randomly in the Health Examination Center.

Study participants, both cases and controls, were also excluded for the following reasons: (a) pregnancy and breastfeeding status; (b) current use of VitD supplements and/or drugs associated with VitD metabolism (e.g., antiepileptic drugs, glucocorticoids); (c) chronic kidney disease (tests for albuminuria, blood creatinine and urea nitrogen); and (d) hypothyroidism or hyperthyroidism. In the first session, all subjects were asked to maintain a stable lifestyle in the 6 months prior to their enrollment in this study. If they answered positively, then they were admitted to the study; otherwise, they were excluded.

### 2.2. Clinical and Laboratory Parameters

After an overnight fast, all measurements and data were collected in the same morning. A structured questionnaire was conducted to obtain information on the participants’ demographics, medical history and lifestyle characteristics, details of which are available in Scheme S1. Notably, alcohol consumption was classified as non-drinker and non-alcoholic drinker (>0 to 210/140 g weekly in men/women). Physical activity included any form of exercise, e.g., walking, running, biking, weight training, sports, stretching and swimming. All participants also received physical examinations to evaluate body weight, height, waist circumference (WC) and blood pressures. Hypertension was diagnosed in participants whose blood pressure levels were ≥ 140/90 mmHg [14]. Metabolic syndrome (MetS) presence was defined according to the modified criteria for Asians established by the International Diabetes Federation Task Force in 2009 [15].

In addition, serum samples were collected to assess liver biochemistry markers, lipid profiles, uric acid (UA), FBG and fasting insulin (FINS). Hypersensitive C-reactive protein (Hs-CRP) was measured using the immunity transmission turbidity method with an AU5800 automatic biochemistry analyzer (Beckman Coulter, Brea, CA, USA). Hepatitis B surface antigen (HBsAg) and hepatitis B envelope antigen (HBeAg) were quantified by chemiluminescence immunoassay (ARCHITECT i2000SR, Abbott Park, IL, USA), and HBV DNA by real-time quantitative polymerase chain reaction (PCR) assay (Roche Cobas TaqMan HBV Test, Roche Diagnostics, Mannheim, Germany). The normal upper limit for ALT was set to 30 U/L for males and 19 U/L for females [16]. HBV DNA was divided into high viral load and low viral load based on 2000 IU/mL. HOMA-IR was calculated as [FINS (µU/mL) × FBG (mmol/L)]/22.5 and its cutoff value, 2.5, was utilized to identify IR [13,17]. Serum VitD levels were measured in the same accredited central laboratory using a Cobas e601 automatic electrochemiluminescence immunoassay analyzer (Roche Diagnostics, Mannheim, Germany). VitD deficiency was defined as VitD ≤20 ng/mL [18].

### 2.3. Radiology Assessments

All participants were preliminarily diagnosed with fatty liver based on abdominal ultrasound using the following criteria: The presence of lower liver-to-kidney contrast with or without the presence of posterior attenuation of the ultrasound beam, intrahepatic vessels blurring and impaired visualization of the gallbladder wall or the diaphragm [19].

Ultrasound-diagnosed fatty liver was subsequently confirmed by magnetic resonance imaging-based proton density fat fraction (MRI-PDFF) with a 3.0-Tesla MRI scanner (Siemens 3.0T MAGNE-TOM Verio; Siemens, Munich, Germany), from which LFC of the entire liver was calculated. The scanning protocol and imaging parameters were the same as described in our previous published study [20]. Steatosis was defined as an average LFC ≥5%, and its severity was graded as follows: mild (<16.3%), moderate (16.3–21.7%) and severe (>21.7%) steatosis, based on the results acquired from the published meta-analysis of [21].

Liver stiffness measurement (LSM) was conducted in all subjects except healthy controls via two-dimensional shear wave elastography (2D-SWE, Aix-en-Provence, France) by two fixed physicians blinded to the clinical information. The valid examination results were based on five eligible acquisitions with an interquartile range (IQR)-to-median ratio less than 0.3 for each participant. The cutoff value of LSM for significant liver fibrosis was set as 7.1 kpa [22].

### 2.4. Histopathological Evaluation

For some of our patients, liver biopsy was performed by an 18G Temno needle in the right hepatic lobe to obtain two biopsy specimens at least 15 mm in length. Histological characteristics were scored based on the NASH clinical research network system and the NAFLD activity scores (NAS), and subclassified as steatosis, lobular inflammation, hepatocyte ballooning and fibrosis [23]. If there were any inconsistencies in scoring, a third pathologist re-evaluated the specimen to achieve a final consensus. A steatosis score of ≥2 was utilized to distinguish those with mild vs. moderate–severe steatosis. A NAS of ≥4 was defined as higher histologic severity for the analyses.

### 2.5. Statistical Analysis

The data analysis was conducted using SPSS statistics software (version 25.0, IBM, Chicago, IL, USA). When continuous variables followed a normal distribution, they are expressed as the mean with standard deviation (SD); otherwise, they are presented as the median with IQR. One-way ANOVA and Kruskal–Wallis tests were utilized to compare continuous variables with Bonferroni adjustments in multi-comparisons. Chi-square tests were used to analyze categorical variables. Pearson’s or Spearman’s correlation analysis as well as multivariate linear regression models were applied to test the associations between VitD and other clinical parameters. Logistic regression analysis was utilized to determine the risk of MAFLD-associated complications (IR, MetS, moderate–severe steatosis and significant fibrosis) for different tertiles of VitD. Two-sided *p* values less than 0.05 were considered significant.

## 3. Results

### 3.1. Subject Characteristics

A total of 1952 consecutive subjects were initially enrolled. We excluded subjects who were pregnant or breastfeeding (*n* = 4), those with alcoholic liver disease (*n* = 33), drug-induced liver injury (*n* = 21), autoimmune hepatitis (*n* = 9), other viral hepatitis (*n* = 13), Wilson’s disease (*n* = 2), those taking either VitD supplements or drugs associated with VitD metabolism (*n* = 43), those with chronic kidney disease (*n* = 16), and those with hypothyroidism or hyperthyroidism (*n* = 24). Thus, 1787 subjects were recruited in the final analysis, including 360 (20.1%) subjects in the healthy control (HC) group, 689 (38.6%) patients in the CHB group, 529 (29.6%) patients in the MAFLD group, and 209 (11.7%) patients in the MAFLD with CHB group. The mean age was 44.2 ± 11.2 years in the entire cohort, with the majority (70.8%) being men. Sex proportions and ages were comparable among the groups.

Serum levels of VitD were highest in the HC group and lowest in the MAFLD group (HC vs. CHB vs. MAFLD with CHB vs. MAFLD, 29.0 ± 9.5 vs. 27.4 ± 9.5 vs. 26.7 ± 8.5 vs. 24.4 ± 8.2 ng/mL, *p*< 0.001, Table 1). Hypertension was most common in the MAFLD with CHB group (38.8%) and the MAFLD group (37.1%), followed by CHB (25.6%) and HC (12.8%). The HC group had lower liver enzyme levels, including ALT, aspartate aminotransferase (AST), γ-glutamyl transpeptidase (GGT) and alkaline phosphatase, than all of the other groups (all *p* < 0.001, Table 1). Compared with healthy controls and with CHB alone, MAFLD patients with and without CHB presented a significantly higher body mass index (BMI), WC, waist–hip ratio, FBG, HOMA-IR, UA, GGT and albumin, as well as a significant difference in serum lipid profiles (all *p* < 0.05, Table 1). Furthermore, subjects with MAFLD alone tended to have higher FBG, UA, Hs-CRP and lipid profiles, except for high-density lipoprotein cholesterol (HDL-C), but lower VitD and AST levels, than those with both MAFLD and CHB (all *p* < 0.05, Table 1). Moreover, regarding HBV viral markers, CHB patients had higher HBV DNA (5.4 ± 1.9 vs. 4.6 ± 2.0 log10 IU/mL, *p* < 0.001) and HBsAg (3.3 ± 1.0 vs. 2.8 ± 1.4 log10 IU/mL, *p* < 0.001) levels than CHB patients with MAFLD, but the prevalence of HBeAg positivity was not significantly different between the two groups (40.3% vs. 32.8%, *p* = 0.057).

Regarding lifestyle characteristics, there were marked differences among the groups except for fatty fish consumption (Table 2). Compared with healthy controls, MAFLD patients with or without CHB had a higher proportion of non-alcoholic drinkers and smokers, and a lower rate of daily exercise and intake of milk (all *p* < 0.001, Table 2). Notably, MAFLD patients had the lowest proportion of individuals spending more than 2 hours outside between sunrise and sunset (*p* < 0.001, Table 2).

### 3.2. Factors Associated with VitD Deficiency

Multivariate analysis revealed that age increased per 5 years (OR 0.73; 95%CI 0.60–0.90, *p* = 0.003), LFC increased per 5% (OR 1.48; 95%CI 1.11–1.97, *p* = 0.008), cold season (OR 2.57; 95%CI 1.20–5.53, *p* = 0.008), time spent outside (OR 0.29; 95%CI 0.10–0.87, *p* = 0.027), and egg (OR 0.26; 95%CI 0.07–0.98, *p* = 0.046) and milk (OR 0.29; 95%CI 0.111–0.77, *p* = 0.013) consumption were independent predictors of VitD deficiency in the MAFLD alone group (Supplementary Table S1). Notably, only time spent outside (OR 0.38; 95%CI 0.15–0.97, *p* = 0.042) was significantly correlated with VitD deficiency in the MAFLD with CHB group (Supplementary Table S1).

### 3.3. Correlation between VitD and Severity of Metabolic, Steatosis and Liver Injuries

**Figure 1:**
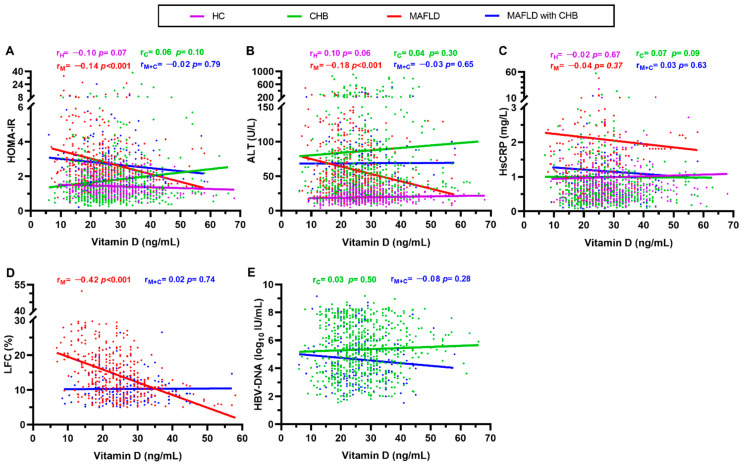
Distribution and correlation between VitD and other clinical parameters. (**A**) Correlation of serum VitD level with homeostasis model assessment of insulin resistance (HOMA-IR) in all four groups. (**B**) Correlation of serum VitD level with alanine aminotransferase (ALT) in all four groups. (**C**) Correlation of serum VitD level with hypersensitive C-reactive protein (Hs-CRP) in all four groups. (**D**) Correlation of serum VitD level with liver fat content (LFC) in the MAFLD and MAFLD with CHB groups. (**E**) Correlation of serum VitD level with hepatitis B virus (HBV) viral load in CHB and MAFLD with CHB groups. HC, healthy control; CHB, chronic hepatitis B; MAFLD, metabolic-associated fatty liver disease; VitD, vitamin D. *r*_H_, *r*_C_, *r*_M_ and *r_M+C_* represent the correlation coefficients in the healthy controls (HC), CHB, MAFLD and MAFLD with CHB groups, respectively.

Serum VitD levels were weakly, but significantly, correlated with ALT (*r* = −0.18, *p* < 0.001, Figure 1A) and HOMA-IR (*r* = −0.14, *p* = 0.001, Figure 1B) in the MAFLD group, whereas no such association was observed in any of the other three groups. Meanwhile, there was a significant inverse correlation between VitD levels and LFC in MAFLD patients (*r* = −0.42, *p* < 0.001) but not in MAFLD with CHB patients (*r* = 0.09, *p* = 0.16, Figure 1D). However, VitD was unrelated to Hs-CRP in all groups (all *p* > 0.05, Figure 1C). No significant correlations were found between VitD levels and HBV DNA (log10 IU/mL) in subjects with CHB (*r* = 0.01, *p* = 0.66) or the co-incidence of CHB and MAFLD (*r* = −0.08, *p* = 0.26, Figure 1E).

Multivariate linear regression in MAFLD-alone patients showed that serum VitD levels were significantly associated with LFC in unadjusted linear regression analysis. After adjustment for the variables included in Models 1, 2 and 3 (age, sex, BMI, WC, recruitment season, ALT, ALB, UA, HOMA-IR, Hs-CRP, Mets, LSM, cigarette and alcohol consumption, time spent outside, the frequency and duration of physical activity, and intake of fatty fish, eggs and milk), VitD was still negatively correlated with LFC (*β* = −0.31, −0.31, −0.38, respectively; all *p* < 0.001, Table 3). However, VitD was not associated with LFC in MAFLD patients with CHB.

We further evaluated the association between VitD and hepatic steatosis by liver histology. Liver biopsy data were available for 54 (10.2%) MAFLD and 18 (8.6%) MAFLD with CHB patients. There were no differences in age or sex between all and biopsy-proven MAFLD patients or all and biopsy-proven MAFLD with CHB patients (all *p* > 0.05, Supplementary Table S2). In patients with MAFLD, marked differences were observed in serum VitD levels between mild and moderate–severe steatosis (25.5 ± 6.6 vs. 19.4 ± 7.1 ng/mL, *p* = 0.002) and between low and high NAS (24.8 ± 6.7 vs. 17.4 ± 7.0 ng/mL, *p* = 0.001), while there were no differences between subjects with or without lobular inflammation, ballooning, and fibrosis, respectively (all *p* > 0.05, Supplementary Figure S1A). For MAFLD with CHB patients, VitD levels showed no significant differences among the subgroups, which were grouped by the grade of steatosis, lobular inflammation, ballooning, fibrosis or NAS (all *p* > 0.05, Supplementary Figure S1B).

### 3.4. Dose-Dependent Relationship of Serum VitD Levels with Disease Severity among Different Groups

**Figure 2:**
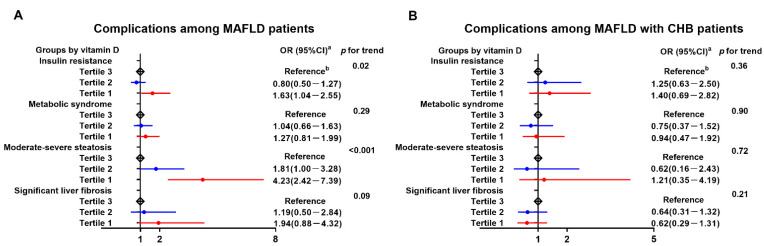
Dose-dependent relationships between VitD tertiles and complications in MAFLD patients without (**A**) or with (**B**) CHB. CHB, chronic hepatitis B; MAFLD, metabolic-associated fatty liver disease; VitD, vitamin D. Insulin resistance is defined as homeostasis model assessment of insulin resistance >2.5. Metabolic syndrome is defined as meeting at least three of the following criteria: (a) waist circumference ≥90 cm (men) and ≥80 cm (women); (b) blood pressure ≥130/85 mmHg or use of hypertensive medication; (c) reduced high-density lipoprotein cholesterol level (<1.03 mmol/L for males, <1.3 mmol/L for females); (d) elevated triglyceride level (≥1.7 mmol/L); and (e) elevated fasting plasma glucose (≥5.6 mmol/L) or drug treatment for hyperglycemia. Significant fibrosis is defined as liver stiffness measurements >7.1 kPa. Moderate–severe steatosis is defined as liver fatty content ≥16.3%.

The role of VitD in the risk of MAFLD-associated disorders is presented in Figure 2. Among the MAFLD or MAFLD with CHB group, serum VitD levels were categorized for 33% (21 and 27 ng/mL, respectively) and 66% (23 and 29 ng/mL, respectively) percentiles, represented as tertile 1 (T1), tertile 2 (T2) and tertile 3 (T3). The VitD T3 in each group was set as a reference. All dose–response associations were adjusted for sex and age. Across the tertiles of VitD levels, there were significant dose-dependent associations between decreased VitD and IR (*p* for trend = 0.02, Figure 2A) and moderate–severe steatosis (*p* for trend < 0.001, Figure 2A) in MAFLD patients; however, such relationships disappeared in CHB with MAFLD patients (*p* for trend = 0.36 and 0.72, respectively; Figure 2B). Moreover, serum VitD had no significant influence on the presence of MetS or significant fibrosis, not only in MAFLD patients (Figure 2A) but also in CHB with MAFLD patients (Figure 2B).

### 3.5. Serum VitD Levels and Different Infection Statuses of CHB Patients with or without MAFLD

**Figure 3:**
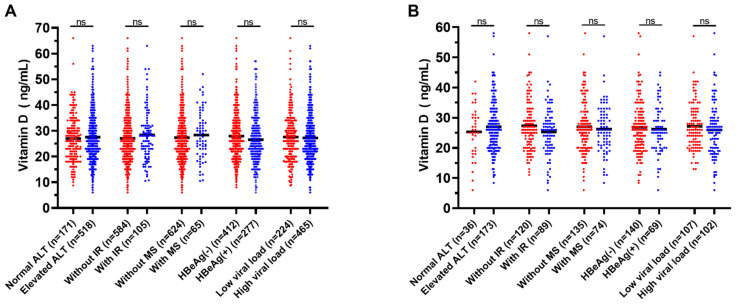
Serum VitD levels in CHB patients with different conditions without (**A**) or with (**B**) MAFLD. CHB, chronic hepatitis B; MAFLD, metabolic-associated fatty liver disease; VitD, vitamin D; ALT, alanine aminotransferase; IR, insulin resistance; MS, metabolic syndrome; HBeAg, hepatitis B envelope antigen. Elevated ALT is defined for males and females as >30 and 19 U/L, respectively. Insulin resistance is defined as homeostasis model assessment of insulin resistance >2.5. Metabolic syndrome is defined as meeting at least three of the following criteria: (a) waist circumference ≥90 cm (males) and ≥80 cm (females); (b) blood pressure ≥130/85 mmHg or use of hypertensive medication; (c) reduced high-density lipoprotein cholesterol level (<1.03 mmol/L for males, <1.3 mmol/L for females); (d) elevated triglyceride level (≥1.7 mmol/L); and (e) elevated fasting plasma glucose (≥5.6 mmol/L) or drug treatment for hyperglycemia. High viral load is defined as HBV DNA level ≥2000 IU/mL.

To further explore the associations between VitD and viral factors, we performed a comparison of VitD levels in different subgroups among CHB patients with or without MAFLD. However, not only in the CHB patients, but also in MAFLD with CHB patients, no significant differences were observed in regard to serum VitD levels among the subgroups that were grouped by the presence of abnormal ALT, IR, Mets, HBeAg-positive or high viral load (all *p* > 0.05, Figure 3).

## 4. Discussion

To our knowledge, this is the first study to evaluate the VitD status of MAFLD with CHB patients, and to investigate whether dose–response associations exist between serum VitD and LFC (determined with MRI-PDFF) independent of lifestyle factors in MAFLD patients with or without CHB. MAFLD with CHB patients presented significantly lower serum VitD levels than age- and sex-matched healthy controls, but higher serum VitD levels than those with MAFLD alone. For MAFLD patients, VitD levels were linearly and negatively correlated with LFC regardless of several confounders, such as HOMA-IR, LSM, MetS and lifestyle factors. However, the abovementioned association was not significant in MAFLD with CHB patients.

Our data show that serum VitD concentrations in MAFLD were 24.4 ± 8.2 ng/mL, which is similar to those reported by Li L et al. and Kim HS et al. (22.1 ± 8.2 and 24.7 ng/mL, respectively) [24,25]. Interestingly, although multiple previous studies have investigated the potential association of VitD with NAFLD, the results have been largely inconsistent. Some reported that VitD levels were independently associated with MAFLD. For example, an Italian study of 262 consecutive subjects revealed that VitD was closely correlated with the fatty liver index regardless of sex, age, IR and MetS [26]. In contrast, another cross-sectional survey of 1248 adults performed in China showed that there was no correlation between VitD and the presence of ultrasound-diagnosed NAFLD [24]. Thus, it is vital to consider the various well-known factors (such as lifestyle habits) influencing serum VitD concentrations, which may account for the inconsistent findings among the different study populations.

In the current study, we attempted to decrease the impact of lifestyle factors, especially sunlight exposure, dietary intake and exercise. Our results show that VitD levels were inversely associated with LFC in a dose-dependent manner independent of lifestyle factors. Notably, the current study first investigated the association between VitD and LFC determined by MRI-PDFF. These findings suggest the value of maintaining normal VitD levels for the prevention and treatment of hepatic steatosis in patients with MAFLD.

Although VitD has been reported to be correlated with IR [26,27], the present study revealed a dose–response association between the stepwise decrease in VitD levels and increased risk of IR in MAFLD subjects; this finding may be because VitD could decrease IR by exhibiting indirect antioxidative properties, reducing inflammation and regulating Ca^2+^ levels in many cell types [6]. Two previous studies carried out on either Chinese or Korean individuals revealed that VitD might be a predictor for MetS in NAFLD [28,29], while our study found that the stepwise decrease in VitD was not correlated with increased risk of MetS in MAFLD subjects. The discrepancies among studies may be caused by the different study populations and methods, the criteria used for VitD classification and/or different approaches to fatty liver and MetS diagnosis.

Moreover, in this study, no clear trend was observed in the association between the lowest tertile levels of VitD and increased risk of significant fibrosis in MAFLD patients, which was in accordance with the published meta-analysis of [30]. However, some researchers have found VitD to be significantly and independently associated with the severity of liver fibrosis in NAFLD. For example, a cross-sectional study from Japan with 229 patients showed that VitD was closely associated with the histological severity of liver fibrosis [31], although this particular study had a different definition for fatty liver than our study. A large prospective population-based survey of 10,960 subjects in the United States also reported that VitD was related to the degree of liver fibrosis; however, they evaluated liver fibrosis by the NAFLD fibrosis score, which is a formula that might compromise the reliability of the results [25]. Thus, with LSM evaluated by 2D-SWE, our findings show a lack of association between VitD and liver fibrosis among Chinese patients with MAFLD.

Contrary to the anticipated findings, the current results found that, interestingly, concomitant CHB had remarkably increased VitD levels compared with MAFLD alone, suggesting a dissimilar association of VitD with MAFLD. Among the MAFLD with CHB patients, our results show that VitD levels were unrelated to LFC and did not differ significantly between subgroups by infection status, including virus replication levels, HBeAg positivity and HBsAg levels. One of the explanations may be that VitD might not be directly associated with glucose and lipid metabolism or virus replication in MAFLD patients with CHB, but the underlying mechanisms have not been clearly elucidated. Another plausible explanation is that CHB patients are more likely to have a healthy lifestyle than MAFLD patients [32]. Our study found a higher level of sunlight exposure in MAFLD patients with CHB.

Previous studies revealed that patients with NAFLD were associated with decreased serum adiponectin levels, whereas those with CHB infection exhibited increased adiponectin levels, indicating a restoration of adiponectin balance in subjects with a combination of the two [33]. Adiponectin is a type of adipokine that can alleviate IR, hepatic steatosis and inflammation, and a positive association between VitD and adiponectin was previously reported [34]. Therefore, upregulated adiponectin might have some bridging association between CHB infection and a reduced risk of MAFLD-associated disorders. Unfortunately, adiponectin was not tested in this study; therefore, further investigations are warranted to provide more information regarding the associations between VitD and adiponectin in MAFLD with CHB patients.

This study had several limitations. First, it had a cross-sectional design that made it difficult to establish a causality nexus. Second, MAFLD was diagnosed using MRI-PDFF instead of liver biopsy, which is the gold standard. However, MRI-PDFF could be a reasonable and relatively accurate alternative method in large-scale studies of the general population because of its noninvasiveness. Third, selection bias might exist in this single-center study. Finally, lifestyle factors were obtained on the basis of self-report questionnaires rather than specific technologies or biomarkers.

In summary, our study reported that patients with concomitant CHB infection had higher VitD levels than those with MAFLD alone, but lower VitD levels than matched healthy controls. Serum VitD levels were inversely associated with LFC in a dose-dependent manner independent of lifestyle factors in subjects with MAFLD, but not in those with MAFLD and CHB. Therefore, the clinical significance of serum VitD concentrations in MAFLD patients with CHB was distinct from that in MAFLD- and CHB-alone patients.

## Figures and Tables

**Table 1:** Demographic and clinical characteristics of enrolled subjects ^1^.

Characteristics	HC (N = 360)	CHB (N = 689)	MAFLD (N = 529)	MAFLD with CHB (N = 209)
Age (years)	45.3 ± 10.5	43.7 ± 11.4	44.3 ± 11.8	43.8 ± 10.5
Male, *n* (%)	249 (69.2%)	472 (68.5%)	388 (73.3%)	157 (75.1%)
BMI (kg/m^2^)	23.0 ± 3.1	22.1 ± 2.9 ** a	26.9 ± 3.3 ** a ** b	26.4 ± 3.3 ** a ** b
WC (cm)	78.5 ± 4.3	78.2 ± 8.3	90.0 ± 7.4 ** a ** b	89.2 ± 8.2 ** a ** b
WHR	0.84 ± 0.03	0.84 ± 0.06	0.90 ± 0.04 ** a ** b	0.89 ± 0.05 ** a ** b
Hypertension ^2^, *n* (%)	46 (12.8%)	176 (25.6%) * a	196 (37.1%) * a * b	81 (38.8%) * a * b
Cold season ^2^, *n* (%)	111 (30.8%)	369 (53.6%) * a	190 (35.9%) * b	109 (52.2%) * a * c
VitD (ng/mL)	29.0 ± 9.5	27.4 ± 9.5 * a	24.4 ± 8.2 ** a ** b	26.7 ± 8.5 * a * c
CHOL (mmol/L)	5.0 ± 1.0	4.8 ± 1.0 * a	5.6 ± 1.1 ** a ** b	5.3 ± 1.1*a ** b * c
TG (mmol/L)	1.0 (0.8, 1.4)	0.9 (0.7, 1.2) ** a	1.9 (1.4, 2.6) ** a ** b	1.4 (1.0, 2.0) ** a ** b ** c
HDL-C (mmol/L)	1.3 ± 0.3	1.3 ± 0.3	1.2 ± 0.3 ** a **b	1.2 ± 0.3 ** a ** b
LDL-C (mmol/L)	3.1 ± 0.7	3.0 ± 0.8 * a	3.5 ± 0.8 **a ** b	3.4 ± 0.9 * a ** b * c
FBG (mmol/L)	4.7 (4.4, 5.1)	4.8 (4.4, 5.2)	5.1 (4.7, 5.7) ** a ** b	4.9 (4.5, 5.5) ** a * b * c
HOMA-IR	1.3 (1.0, 1.7)	1.3 (0.9, 2.0)	2.4 (1.6, 3.4) ** a ** b	2.3 (1.5, 3.3) ** a ** b
UA (μmol/L)	365 ± 94	344 ± 96 * a	435 ± 103 ** a ** b	404 ± 92 ** a ** b * c
ALT (U/L)	18.0 (13.3, 24.0)	45.0 (26.0, 86.5) ** a	42.0 (25.0, 75.5) ** a	49.0 (31.0, 80.5) ** a
AST (U/L)	21.0 (18.0, 25.0)	38.0 (26.0, 65.0) ** a	29.0 (22.0, 43.8) ** a ** b	34.0 (26.0, 49.0) ** a ** c
GGT (U/L)	20.5 (15.0, 29.0)	28.0 (19.0, 50.0) ** a	42.0 (29.0, 73.0) ** a ** b	36.5 (24.8, 69.3) ** a ** b
ALP (U/L)	70.0 (61.0, 80.0)	79.0 (66.0, 94.0) ** a	76.0 (67.0, 89.0) ** a	75.0 (65.0, 89.0) ** a
ALB (g/L)	43.6 ± 3.3	43.4 ± 4.1	45.1 ± 3.0 ** a ** b	44.6 ± 3.2 * a ** b
TB (μmol/L)	12.9 (10.4, 16.7)	14.9 (11.6, 20.4) ** a	13.5 (10.8, 16.8) ** b	13.9 (11.2, 17.9)
Hs-CRP (mg/L)	0.7 (0.4, 1.1)	0.6 (0.3, 1.0) * a	1.4 (0.7, 3.0) **a ** b	0.8 (0.4, 1.5) ** b ** c

Note. Abbreviations: HC, healthy control; CHB, chronic hepatitis B; MAFLD, metabolic-associated fatty liver; BMI, body mass index; WC, waist circumference; WHR, waist–hip ratio; VitD, vitamin D; CHOL, total cholesterol; TG, triglycerides; HDL-C, high-density lipoprotein cholesterol, LDL-C, low-density lipoprotein cholesterol; FBG, fasting blood glucose; HOMA-IR, homeostasis model assessment of insulin resistance; ALT, alanine aminotransferase; AST, aspartate aminotransferase; GGT, γ-glutamyl transpeptidase; ALP, alkaline phosphatase; UA, uric acid; ALB, albumin; TB, total bilirubin; Hs-CRP, hypersensitive C-reactive protein. ^1^ Values are expressed as mean ± SD, median (IQR) and *n* (%). a—compared with HC group, b—compared with CHB group, c—compared with MAFLD group; * *p* < 0.05, ** *p* < 0.001. ^2^ Hypertension, those with average blood pressure levels ≥140/90 mmHg or use of hypertensive medication; cold season denotes September to February.

**Table 2:** Lifestyle characteristics of the population ^1^.

Characteristics	HC (N = 360)	CHB (N = 689)	MAFLD (N = 529)	MAFLD with CHB (N = 209)	*p*
Non-alcoholic drinker ^2^	73 (20.3%) ^a^	139 (20.2%) ^a^	163 (30.8%) ^b^	70 (33.5%) ^b^	<0.001
Smoker ^2^	25 (6.8%) ^a^	103 (14.9%) ^b^	101 (19.1%) ^b^	37 (17.7%) ^b^	<0.001
Low education level ^2^	194 (54.0%) ^abc^	376 (54.6%) ^c^	225 (42.5%) ^b^	126 (60.3%) ^ac^	<0.001
Time outside between sunrise and sunset					<0.001
<1 h/day	121 (33.6%) ^a^	271 (39.4%) ^ab^	228 (43.1%) ^b^	71 (34.0%) ^ab^	
1–2 h/day	113 (31.4%) ^ab^	169 (24.5%) ^b^	183 (34.6%) ^a^	63 (30.1%) ^ab^	
≥2 h/day	126 (35.0%) ^a^	249 (36.1%) ^a^	118 (22.3%) ^b^	75 (35.9%) ^a^	
Sun protection					<0.001
Usually (≥50%)	31 (8.6%) ^a^	153 (22.2%) ^b^	51 (9.6%) ^ac^	33 (15.9%) ^bc^	
Sometimes (<50%)	24 (6.7%) ^a^	143 (20.7%) ^b^	100 (18.9%) ^b^	28 (13.5%) ^b^	
Never	305 (84.7%) ^a^	393 (57.1%) ^b^	378 (71.5%) ^c^	147(70.5%) ^c^	
Frequency of physical activity					<0.001
Never	101 (28.1%) ^a^	256 (37.2%) ^b^	250 (47.3%) ^c^	109 (52.2%) ^c^	
1/week	15 (4.2%) ^a^	57 (8.3%) ^a^	38 (7.2%) ^a^	17 (8.2%) ^a^	
2–4/week	137 (38.1%) ^a^	241 (35.0%) ^a^	146 (27.6%) ^b^	50 (23.7%) ^b^	
5–6/week	26 (7.2%) ^a^	82 (11.9%) ^a^	60 (11.3%) ^a^	25 (12.1%) ^a^	
Every day	81 (22.5%) ^a^	53 (7.7%) ^b^	35 (6.6%) ^b^	8 (3.9%) ^b^	
Duration of physical activity					<0.001
Never	101 (28.1%) ^a^	256 (37.2%) ^b^	250 (47.3%) ^c^	109 (52.2%) ^c^	
<3 h/week	92 (25.6%) ^a^	263 (38.2%) ^b^	165 (31.2%) ^ab^	61 (29.0%) ^ab^	
≥3 h/week	167 (46.4%) ^a^	170 (24.7%) ^b^	114 (21.6%) ^b^	39 (18.8%) ^b^	
Fatty Fish (≥1 x/week)	100 (27.8%) ^a^	192 (27.9%) ^a^	154 (29.1%) ^a^	50 (23.7%) ^a^	0.53
Liver (≥1 x/week)	77 (21.4%) ^a^	94 (13.7%) ^b^	86 (16.3%) ^ab^	21 (10.1%) ^b^	0.001
Margarine (≥1 x/week)	29 (8.1%) ^a^	10 (1.5%) ^b^	7 (1.3%) ^b^	2 (1.0%) ^b^	<0.001
Eggs					<0.001
Never	108 (30.0%) ^a^	196 (28.5%) ^a^	62 (11.7%) ^b^	65 (30.9%) ^a^	
1–6 units/week	95 (26.4%) ^a^	372 (54.0%) ^b^	303 (57.3%) ^b^	102 (48.8%) ^b^	
≥7 units/week	157 (43.6%) ^a^	121 (17.5%) ^b^	164 (31.0%) ^c^	42 (20.3%) ^b^	
Milk					<0.001
Never	184 (51.1%) ^a^	503 (73.0%) ^b^	326 (61.6%) ^c^	142 (68.1%) ^bc^	
Sometimes (≥1 x/week)	82 (22.8%) ^a^	121 (17.5%) ^a^	118 (22.3%) ^a^	43 (20.3%) ^a^	
Every day	94 (26.1%) ^a^	65 (9.5%) ^b^	85 (16.1%) ^c^	24 (11.6%) ^bc^	

Note. Abbreviations: HC, healthy control; CHB, chronic hepatitis B; MAFLD, metabolic-associated fatty liver. ^1^ Data are presented as *n* (%). Each subscript letter (e.g., a, b, c) denotes a subset of group categories whose column proportions do not differ significantly from each other at the 0.05 level. ^2^ Non-alcoholic drinker is defined for males/females as >0 to 210/140 g weekly; smoker is defined as ≥1 cigarette/day; low education level denotes middle school, primary school or lower.

**Table 3:** Multivariate regression analysis for the associations between vitamin D and liver fat content in MAFLD patients with and without CHB.

	Liver Fat Content ^1^					
	MAFLD			MAFLD with CHB		
	β	95% CI	*p*	β	95% CI	*p*
Unadjusted	−0.352	(−0.417, −0.287)	<0.001	−0.016	(−0.097, 0.064)	0.69
Model 1 ^2^	−0.276	(−0.344, −0.209)	<0.001	-	-	-
Model 2 ^3^	−0.280	(−0.346, −0.214)	<0.001	-	-	-
Model 3 ^4^	−0.353	(−0.434, −0.272)	<0.001	-	-	-

^1^ Liver fat content as the dependent variable. ^2^ Model 1: adjusted for age, gender, BMI, WC, recruitment season. ^3^ Model 2: model 1 plus ALT, ALB, UA, HOMA-IR, Hs-CRP, MetS and LSM. ^4^ Model 3: model 2 plus cigarette and alcohol consumption, time spent outside between sunrise and sunset, the frequency and duration of physical activity, and fatty fish, egg and milk consumption.

## Data Availability

Data supporting the reported results and conclusions are available from the corresponding author on reasonable request.

## Supplementary Materials

The following supporting information can be downloaded at: https://www.mdpi.com/article/10.3390/nu14102114/s1, Scheme S1: Questionnaire on personal characteristics of the participants; Table S1: Factors associated with vitamin D deficiency in patients with different liver diseases; Table S2: Comparison of baseline characteristics between all and biopsy-proven MAFLD, and all and biopsy-proven MAFLD with CHB, respectively; Figure S1: Comparison of serum vitamin D concentrations across categorized liver histological features in MAFLD group (A) and MAFLD with CHB group (B), respectively.
