# Supplementary material for: Vitamin D Status Presents Different Relationships with Severity in Metabolic-Associated Fatty Liver Disease Patients with or without Hepatitis B Infection
Source: Nutrients. 2022 May 18;14(10):2114. doi: 10.3390/nu14102114 (PMC9147199; doi:10.3390/nu14102114)
Supplement: Supplementary file 1 [file nutrients-14-02114-s001.zip › nutrients-1698374-supplementary.pdf]

Supplementary

Scheme S1: Questionnaire on personal characteristics of the participants:

Identification Number: ____________________

Time of enrollment (DD/MM/YY):____________________

Part 1. Demographic characteristics

1.1 Name:________________

1.2 Gender: male/female

1.3 Age:________________ in years; Date of birth (DD/MM/YY):________________

1.4 Address: City _______________; Sub-city _______________; Woreda _______________

1.5 Phone number: ________________; Telephone: ________________

1.6 Education status: (1) Illiteracy / (2) Primary school / (3) Middle school / (4) Junior college / (5) University and above

1.7 Occupation: Employed /Unemployed, specify ___________________________________

If employed, the main workplace: Indoor /Outdoor /Both indoor and outdoor

1.8 Number of family members: __________________________

Part 2. Medical history

2.1 History of present illness: ____

If yes, when was it diagnosed? __________weeks; where was it diagnosed? _____________; what drug were you taking? ________________

2.2 Previous medical history: ____

If yes, when was it diagnosed? _____________weeks; where was it diagnosed? ______________; what drug were you taking? ________________

2.3 The history of surgery and blood transfusion: ___________________________________

2.4 The history of smoking: Never or past /current (<10 cigarettes a day/10－20 cigarettes a day/>20 cigarettes a day), smoking for_______years, quitting for smoking for _______years

2.5 The history of drinking: Never or past /current; drinking for________years， ________g alcohol /week，abstinence for ________years

2.6 Family history:

Hepatitis B: Yes /No, father /mother /brothers and sisters /children ______________________

Fatty liver: Yes /No, father /mother /brothers and sisters /children _______________________

Obesity: Yes /No, father /mother /brothers and sisters /children _________________________

Hypertension: Yes /No, father /mother /brothers and sisters /children _____________________

Diabetes mellitus: Yes /No, father /mother /brothers and sisters /children __________________

Coronary heart disease: Yes /No, father /mother /brothers and sisters /children _____________

Others:____

Part 3. lifestyle characteristics

3.1 How much time is spent outside between sunrise and sunset on a routine day?

A <1 hour/day

B 1-2 hours/day

C >2 hours/day

3.2 What is the frequency of sun protection when outdoors? (Sun protection denoted that exposed skin was covered using sunscreen or clothing)

A Never

B Sometimes(<50%), specify _________________

C Usually (≥50%), specify _________________

3.3 What is the frequency of physical activity in a typical week? (Examples of physical activities included walking, running, biking, weight training, sports, stretching and swimming)

A Never

B 1 day/week

C 2-4 day/week

D 5-6 day/week

E everyday

3.4 What is the usual duration of physical activity session?

A Never

B <0.5 hour/day

C 0.5-1 hour/day

D 1-2 hours/day

E 2-3hours/day

F >3 hours/day

3.5 Do you take vitamin D supplements or cod liver oil?

A No

B Yes, specify ________________

3.6 Do you eat fatty fish at least once a week? (Examples of fatty fishes included salmon, largehead hairtail, grouper, corvina, lingcod, yellow croaker, eel and silver sillago)

A No

B Yes, specify ________________

3.7 Do you eat liver at least once a week?

A No

B Yes, specify ________________

3.8 Do you eat margarine at least once a week?

A No

B Yes, specify ________________

3.9 How many eggs do you eat in a typical week?

A None

B 1-6 units /day

C ≥7 units /day

3.10 How often do you drink milk in a typical week?

A Never

B Sometimes (at least once a week), specify ________________

C Everyday, specify ________________

Table S1. Factors associated with vitamin D deficiency [1] in patients with different liver diseases [2].

Factors	CHB			MAFLD			MAFLD with CHB		
	Univariate	Multivariate		Univariate	Multivariate		Univariate	Multivariate	
	OR	*P*	OR (95%CI)	OR	*P*	OR (95%CI)	OR	*P*	OR (95%CI)
Male	0.35 **	0.001	0.37(0.21-0.66)	0.55 *	0.16		0.65		
Age increased per 5 years	0.89 *	0.007	0.83(0.73-0.95)	0.78 **	0.003	0.73(0.60-0.90)	0.93		
Obesity [3]	0.69			1.23			0.65		
Increased WC [3]	0.98			1.41			0.87		
Hypertension [3]	0.99			0.87			1.75^{+}	0.11	
CHOL >5.2mmol/L	0.70^{+}	0.78		0.64 *	0.31		0.90		
TG >1.7mmol/L	0.70			0.77			0.55^{+}	0.45	
LDL-C >3.4mmol/L	0.54 *	0.47		0.70^{+}	0.16		1.02		
HOMA-IR >2.5	0.79			2.08 *	0.94		1.21		
Hyperuricemia [3]	0.65^{+}	0.48		0.76			0.99		
ALT elevation [3]	0.83			1.48^{+}	0.19		0.68		
Hs-CRP (mg/L)	0.90			0.98			0.98		
LFC increased per 5%	-			1.47 **	0.008	1.48(1.11-1.97)	1.01		
Lg_{10}(HBV-DNA)	0.93			-			1.16		
HBeAg (positive)	1.14			-			1.07		
Cold season [3]	1.36			1.88 *	0.016	2.57(1.20-5.53)	1.16		
Non-alcoholic drinker [3]	0.57 *	0.37		0.46 *	0.82		0.68		
Smoker [3]	0.35 *	0.10		0.33 *	0.68		1.39		
Low education level [3]	0.67^{+}	0.23		1.00			1.11		
Time outside (sunrise to sunset)									
< 1h/day	reference			reference			reference		
1-2 hrs/day	0.62			0.66			0.61		
≥ 2hrs/day	0.18 **	<0.001	0.22(0.11-0.45)	0.39 *	0.027	0.29(0.10-0.87)	0.36 *	0.042	0.38(0.15-0.97)
Sun protection									
Never	reference			reference			reference		
Sometimes	1.10			0.95			0.71		
Usually (≥50%)	1.58			1.42			1.40		
Frequency of physical activity									
Never	reference			reference			reference		
1 per week	1.58			0.87			1.61		
2-4 per week	1.03			0.59			0.78		
5-6 per week	0.64			0.65			1.93		
Every day	0.23^{+}	0.14		0.24^{+}	0.28		1.29		
Duration of physical activity									
Never	reference			reference			reference		
<3hrs/week	1.23			0.71			1.11		
≥3hrs/week	0.47 *	0.12		0.42 *	0.36		1.29		
Fatty Fish (≥1x/week)	0.77			0.79			0.68		

Liver (≥1x/week)	1.23	0.89			1.17
Eggs					
Never	reference	reference			reference
1-6 units/week	1.49	0.35*	0.08		1.82
≥7 units/week	1.04	0.38*	0.046	0.26(0.07-0.98)	1.64
Milk					
Never	reference	reference			reference
Sometimes (1-6x/week)	0.89	0.46 *	0.013	0.29(0.11-0.77)	1.92
Every day	0.65	0.86			1.53

Abbreviations: CHB, chronic hepatitis B; MAFLD, metabolic associated fatty liver; WC, waist circumference; ALT, alanine aminotransferase; CHOL, total cholesterol; TG, triglycerides; LDL-C, low-density lipoprotein cholesterol; HOMA-IR, homeostasis model assessment of insulin resistance;Hs-CRP, hypersensitive C-reactive protein; LFC, liver fat content; HBV-DNA, hepatitis B virus DNA; HBeAg, hepatitis B envelope antigen. [1] Vitamin D deficiency was defined as: vitamin D ≤20ng/mL. [2] *P* values were for the logistic regression analysis, ^{+}P<0.01, * *P*<0.05, ** *P*<0.001. [3] Obesity was defined as body mass index ≥25 kg/m^2; Hypertension was defined as average blood pressure levels ≥140/90 mmHg or use of hypertensive medication; Increased WC was defined as waist circumference >90 cm for male and >80 cm for female; ALT elevation was defined as male and female at >30 and 19 U/L, respectively; Hyperuricemia was defined as serum uric acid >420 μmol/L for male and >360 μmol/L for female; Cold season denotes September to February; Non-alcoholic drinker was defined as males/females >0 to 210/140g weekly; Smoker was defined as ≥1 cigarette/day; Low education level denotes middle school, primary school, or less.

Table S2. Comparison of baseline characteristics between all and biopsy-proven MAFLD, all and biopsy-proven MAFLD with CHB, respectively [1].

Characteristics	MAFLD			MAFLD with CHB		
	All (N=529)	Biopsy-proven (N=54)	*p*	All (N=209)	Biopsy-proven (N=18)	*p*
Age (years)	44.3±11.8	41.0±11.8	0.053	43.8±10.5	43.8±8.5	0.99
Male, n (%)	388(73.3%)	38(70.4%)	0.64	157(75.1%)	14(77.8%)	0.80
BMI (kg/m2)	26.9±3.3	26.3±3.1	0.22	26.4±3.3	26.2±3.8	0.75
WC (cm)	90.0±7.4	88.4±8.7	0.19	89.2±8.2	88.5±8.9	0.74
WHR	0.90±0.04	0.90±0.05	0.82	0.89±0.05	0.89±0.05	0.98
Hypertension [2], n (%)	196(37.1%)	25(46.3%)	0.19	81(38.8%)	6(33.3%)	0.65
Cold season [2], n (%)	190(35.9%)	21(38.9%)	0.67	109(52.2%)	11(61.1%)	0.47
Vitamin D (ng/mL)	24.4±8.2	22.9±7.4	0.19	26.7±8.5	27.2±7.6	0.82
CHOL (mmol/L)	5.6±1.1	5.3±1.1	0.08	5.3±1.1	5.3±1.1	0.98
TG (mmol/L)	1.9(1.4,2.6)	1.9(1.4,2.4)	0.52	1.4(1.0,2.0)	1.3(0.9,1.8)	0.46
HDL-C (mmol/L)	1.2±0.3	1.2±0.4	0.54	1.2±0.3	1.2±0.2	0.57
LDL-C (mmol/L)	3.5±0.8	3.3±0.8	0.04	3.4±0.9	3.4±0.8	0.999
FBG (mmol/L)	5.1(4.7,5.7)	5.1(4.7,6.0)	0.64	4.9(4.5,5.5)	5.0(4.8,5.3)	0.36
HOMA-IR	2.4(1.6,3.4)	2.6(1.9,4.1)	0.10	2.3(1.5,3.3)	2.3(1.2,3.6)	0.84
UA (μmol/L)	435±103	434±102	0.97	404±92	380±74	0.28
ALT (U/L)	42.0(25.0,75.5)	73.5(43.8,127.0)	<0.001	49.0(31.0,80.5)	46.5(33.5,69.8)	0.73
AST (U/L)	29.0(22.0,43.8)	46.0(33.0,60.3)	<0.001	34.0(26.0,49.0)	33.0(27.0,41.5)	0.93
GGT (U/L)	42.0(29.0,73.0)	64.0(43.8,121.8)	<0.001	36.5(24.8,69.3)	37.0(20.8,62.3)	0.60
ALP (U/L)	76.0(67.0,89.0)	80.5(73.0,88.3)	0.053	75.0(65.0,89.0)	75.5(71.0,83.5)	0.997
ALB (g/L)	45.1±3.0	45.2±3.3	0.86	44.6±3.2	45.9±3.4	0.12
TB (μmol/L)	13.5(10.8,16.8)	13.4(10.7,18.0)	0.89	13.9(11.2,17.9)	14.6(11.7,19.5)	0.45
Hs-CRP (mg/L)	1.4(0.7,3.0)	1.3(0.8,3.1)	0.92	0.8(0.4,1.5)	0.8(0.7,2.2)	0.38
Steatosis grade			0.74			0.42
Mild	317(59.9%)	31(57.4%)		184(88.0%)	14(77.8%)	
Moderate and severe	212(40.1%)	23(42.6%)		25(12.0%)	4(22.2%)	

Abbreviations: MAFLD, metabolic associated fatty liver; CHB, chronic hepatitis B; BMI, body mass index; WC, waist circumference; WHR, waist-hip ratio; CHOL, total cholesterol; TG, triglycerides; HDL-C, high-density lipoprotein cholesterol, LDL-C: low-density lipoprotein cholesterol; FBG, fasting blood glucose; HOMA-IR, homeostasis model assessment of insulin resistance; ALT, alanine aminotransferase; AST, aspartate aminotransferase; GGT, γ-glutamyl transpeptidase; ALP, alkaline phosphatase; UA, uric acid; ALB, albumin; TB, total bilirubin; Hs-CRP, hypersensitive C-reactive protein. 1 Values are expressed as mean±SD, median (IQR) and n (%). 2 Hypertension, those with average blood pressure levels ≥140/90 mmHg or use of hypertensive medication; cold season denotes September to February.

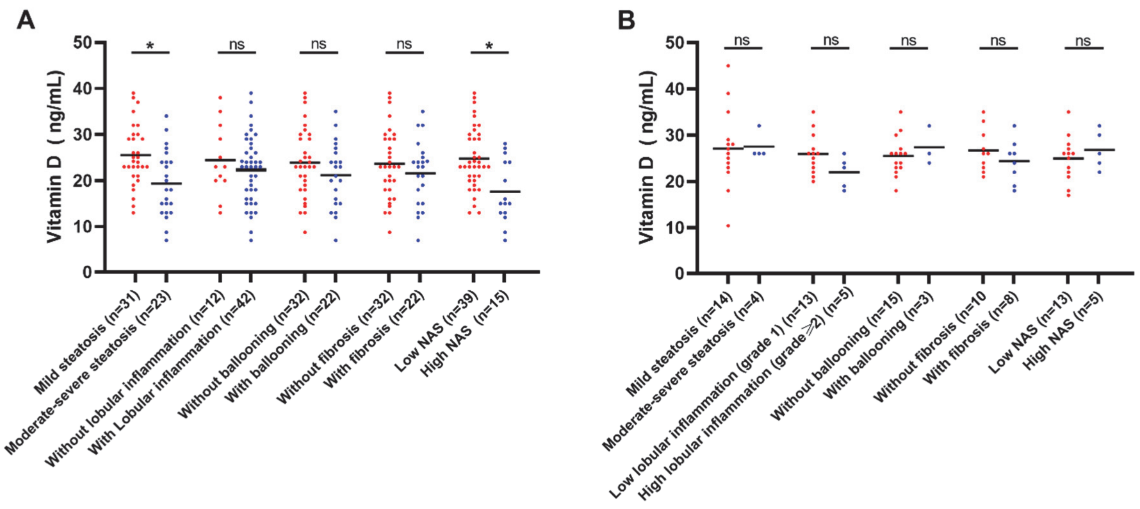

Figure S1. Comparison of serum vitamin D concentrations across liver histological features categories in MAFLD group (**A**) and MAFLD with CHB group (**B**), respectively. CHB, chronic hepatitis B; MAFLD, metabolic associated fatty liver disease. NAS, NAFLD activity scores. Moderate-severe steatosis was defined as the grade of steatosis ≥2. High NAS was defined as NAFLD activity scores ≥4. * $p < 0.05$
